# Lithium Transport Studies on Chloride-Doped Argyrodites as Electrolytes for Solid-State Batteries

**DOI:** 10.1021/acsami.3c10857

**Published:** 2023-11-03

**Authors:** Dominika A. Buchberger, Piotr Garbacz, Krzysztof Słupczyński, Artur Brzezicki, Maciej Boczar, Andrzej Czerwiński

**Affiliations:** † Faculty of Chemistry, University of Warsaw, Pasteura 1, 02-093 Warsaw, Poland; ‡ Adamed Pharma SA, 05-152 Pieńków, Poland

**Keywords:** NMR, EIS, activation energy, Li conductivity, solid-state electrolyte

## Abstract

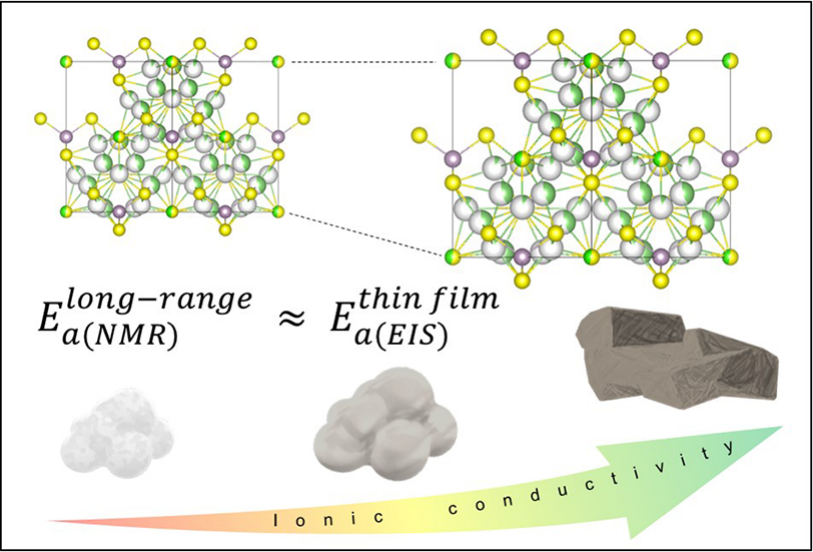

In this study, the activation energy and ionic conductivity of the Li_6_PS_5_Cl material for all-solid-state batteries were investigated using solid-state nuclear magnetic resonance (NMR) spectroscopy and electrochemical impedance spectroscopy (EIS). The results show that the activation energy values estimated from nuclear relaxation rates are significantly lower than those obtained from impedance measurements. The total ionic conductivities for long-range lithium diffusion in Li_6_PS_5_Cl calculated from EIS studies depend on the crystal size and unit cell parameter. The study also presents a new sample preparation method for measuring activation energy using temperature-dependent EIS and compares the results with the solid-state NMR data. The activation energy for a thin-film sample is equivalent to the long-range lithium dynamics estimated from NMR measurements, indicating the presence of additional limiting processes in thick pellets. Additionally, a theoretical model of Li-ion hopping based on results obtained using density-functional theory methods in comparison with experimental findings was discussed. Overall, the study emphasizes the importance of sample preparation methods in determining accurate activation energy and ionic conductivity values for solid-state lithium batteries and the significance of solid-state electrolyte thickness in new solid-state battery design for faster Li-ion diffusion.

## Introduction

Solid-state electrolytes have received broad attention in recent years. This is due to the growing demand on various energy storage devices in numerous portable and automotive applications. There are high expectations of novel lithium-ion battery designs, especially in terms of their higher energy density and higher safety. Solid-state electrolytes hope to reveal these features. The race to create new and safe lithium cells, as well as the chemical–physical and technological problems encountered, causes a growing interest in basic research of battery components and the search for more sophisticated techniques to analyze them.

There are several electrolyte materials classes under intensive investigation. Lithium argyrodites are one of the electrolyte types extensively studied in terms of their stability, conductivity, and performance in prototype cells. They owe their popularity mainly due to one of the highest Li ionic conductivities among inorganic solid electrolytes. There are several synthesis methods that can provide a suitable crystal structure and therefore mechanochemical synthesis^1,2^ or wet-chemical methods. Among those, the wet-chemical method can provide a material in the shortest period and with an acceptable ionic conductivity.^3−5^

The ionic conductivity of solid-state electrolytes is usually calculated based on the electrochemical impedance spectroscopy (EIS) measurements performed on the simplest two-electrode symmetric cell setup. In such a measurement, the compressed pellet is located between two ion-blocking electrodes under external pressure.^6,7^ The ionic conductivity (σ_ion_) that is extracted from that examination is given by
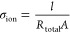
1where *R*_total_ is the total electrical resistance of the solid-state electrolyte (Ω) and *l* and *A* are the pellet’s thickness (cm) and area (cm^2^), respectively.^7^ The total resistance that is used for calculation consists of all measured pellet resistances, typically simplified as ionic resistance of the grain (bulk resistance, *R*_b_) and grain-boundary resistance, *R*_gb_.^6,7^ For sulfide-based solid electrolytes, the resistances *R*_b_ and *R*_gb_ can be challenging to resolve, as they strongly overlay. The semicircle resolution in Nyquist plots can be attempted in low temperatures, since the separation of the grain and the grain boundary resistance semicircles can be distinguished and well fitted.^8^

The ionic conductivity is temperature-dependent and typically shows the Arrhenius behavior; i.e., the relationship of ionic conductivity σ_ion_ and temperature *T* is
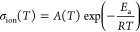
2where, *E*_a_ is the activation energy in joules per a mol of ions, *T* is the temperature in kelvin, *R* ≈ 8.314 J/(mol·K) is the ideal gas constant, and *A*(*T*) is a model-dependent prefactor.^7^ Once the system is operated in a temperature-controlled environment, the activation energy can be measured using electrochemical impedance spectroscopy. EIS measurements provide the total resistance of the materials; therefore, the obtained activation energy is a sum of contributions of all process types taking part in the ionic conduction. Separation of individual contributions from their sum may give a deeper insight into the material conductivity, but modeling individual resistance contributions is complex or sometimes even impossible.

Chloride-doped lithiated argyrodites are known to have one of the highest ionic conductivities among sulfide-based solid-state electrolytes. The experimental and calculated ionic conductivity and activation energy of Li_6_PS_5_Cl material (abbreviated as LPSC) reported in the literature are given in Table 1 (for extanded information, see Table S1).^2,4,5,9−20^ Although these selected papers do not cover the entire research on Li_6_PS_5_Cl solid electrolytes, they do represent the most common preparation techniques and measurement approaches currently used in the field.^21^

**Table 1:** Experimental Total Ionic Conductivity at Room Temperature (298 K) and Calculated Activation Energy of Li_6_PS_5_Cl Material

ref	material preparation method	ionic conductivity measurements	total ionic conductivity (mS cm^–1^)	activation energy (eV)
Solid-State Methods				
(2)	Mechanical milling: Li_2_S, P_2_S_5_, LiCl; 550 °C	EIS, 10 mHz to 200 kHz	0.45	0.33
(9)	Mechanical milling: Li_2_S, P_2_S_5_, LiCl; no annealing	EIS, 10 Hz to 1 MHz	1.3	0.17
(10)	Mechanical milling: Li_2_S, P_2_S_5_, LiCl; 550 °C	EIS, 1 Hz to 10 MHz	0.033 (BM)	0.38
			0.74 (BMA)	0.11
(11)	Mechanical milling: Li_2_S, P_2_S_5_, LiCl; 550 °C	EIS, 10 mHz to 10 MHz	3.8	0.396
		NMR, ^7^Li, 7 T, 116 MHz	9.0	0.17
				0.32
(12)	Mechanical milling: Li_2_S, P_2_S_5_, LiCl; 550 °C	EIS, 100 mHz to 1 MHz	2.5	0.34
(13)	(SSM) Mechanical milling: Li_2_S, P_2_S_5_; 550 °C	EIS, 1 Hz to 1 MHz, amp. 10 mV	5.99 (SSM)	0.34 (SSM, EIS)
		ssNMR, ^7^Li, 155.506 MHz		0.16 (NMR_local_)
				0.19 (NMR_long_)
	(BM) Mechanical milling: Li_2_S, P_2_S_5_; 550 °C	EIS, 1 Hz to 1 MHz, amp. 10 mV	3.25 (BMA)	0.35 (BMA, EIS)
		ssNMR, ^7^Li, 155.506 MHz		0.09 (NMR_local_)
				0.29 (NMR_long_)
(14)	Mechanical milling: Li_2_S, P_2_S_5_, LiCl; 550 °C	ssNMR, ^7^Li, 155.506 MHz	1.18	0.33 (NMR_bulk_)
(15)	purchased from NEI Corporation	EIS, 100 mHz to 1 MHz, amp. 10 mV	3.4	0.28
		Temp-dependent static ^7^Li NMR, 77.8 MHz	3.9 (NMR)	0.14 (NMR_local_)
				0.27 (NMR_long_)
(20)	Mechanical milling: Li_2_S, P_2_S_5_, LiCl; 4h, 550 °C:	EIS, 10 Hz to 1 MHz	6.11	0.3
	(1) powder pellet			
	(2) sintered pellet		3.50	0.33
Wet Chemical Methods				
(4)	Wet chemical synthesis: Li_2_S, Li_3_PS_4_, and LiCl:EtOH, 200 °C	EIS, 100 mHz to 1 MHz, amp. 100 mHz	0.38	0.399
(5)	Wet chemical synthesis: Li_2_S, LiCl, β-Li_3_PS_4_·3THF/THF; EtOH, 140 °C	EIS, 10 mHz to 1 MHz	2.4	
(16)	(1) Mechanical milling	EIS, 100 mHz to 1 MHz	1.4 (MM)	0.23
	(2) Ethanol dissolution: EtOH; 80 °C		0.014 (EtOH)	0.34
(17)	Wet chemical synthesis: Li_2_S, P_2_S_5_ and LiCl: EtOH; 450 °C	EIS, 100 mHz to 3 MHz	0.21	0.5
(18)	(1) Mechanical milling: 600 rpm, 40 h	EIS, 200 Hz to 100 kHz	0.04 (MM)	
	(2) Dissolution: EthOH and ethyl acetate; 150 °C		0.06 (EtOH)	
(19)	Wet chemical synthesis: P_2_S_5_, LiCl, Li_2_S; pyridine; 550 °C	EIS, 1 Hz to 7 MHz	2.6	

The other approach to studying conductivity due to lithium mobility offers nuclear magnetic resonance (NMR) spectroscopy which, among chosen experimental methods for the determination of ionic conductivity and activation energy, is noticeably infrequently used due to the equipment availability and complexity. In NMR studies, usually the lithium-7 isotope is investigated since one takes advantage of its high natural abundance (more than 90%) and relatively high resonance frequency (nearly 40% of the ^1^H frequency), although spin 3/2 of lithium-7, and consequently quadrupole moment, results in some line broadening. NMR studies of lithium ion dynamics across different materials classes are reviewed in ref (22). The insight into how fast the lithium ion moves provides studies of the temperature dependence of the longitudinal relaxation time (*T*_1_). The rigorous derivation of the relationship between the relaxation rate (an inverse of *T*_1_), spin system parameters, and atomic/molecular motions is intractable; therefore, introducing severe approximations is indispensable.^23^ One of them is that the rate of nuclear relaxation is expressed by the autocorrelation function, which quantifies the rate of loss of information about the system’s initial state.^24,25^ Assuming that the rate of lithium atoms jumps between successive positions in the crystal follows the Arrhenius equation (increase with temperature as  where *k*_B_ is the Boltzmann constant), one finds that a plot of 1/*T*_1_ over 1/*T* allows us to find the energy *E*_a_ and the rate of hopping, which if the structure of the crystal containing is known provides conductivity within a microcrystal (a grain) indirectly. Interestingly, the *E*_a_ values estimated from NMR are typically lower and ionic conductivities are higher than those calculated from the EIS technique for the same sample. There is an ongoing discussion on how to explain that phenomenon. Moreover, an inspection of the relaxation rate’s shape in the reciprocal temperature function gives at least qualitative information about the autocorrelation function. For instance, a symmetric shape indicates an exponential decrease of the autocorrelation function, while as is in the case of Li_6_PS_5_Cl, an asymmetric shape may result from reduced dimensionality of the allowed diffusion paths of lithium and the appearance of percolation phenomena.^11,14,26^

In this work, we present a comprehensive study on the activation energy of lithium argyrodite materials calculated using EIS and NMR spectroscopy. For this study, we have chosen the Li_6_PS_5_Cl material synthesized using a wet-chemical method and annealed at temperatures from 200 to 500 °C; the materials prepared in that way were not studied before using NMR spectroscopy. We also demonstrate a new sample preparation method for measuring temperature-dependent EIS and compare the results with NMR studies. Additional structural and morphological data are applied to reveal the nature of the conduction and activation energy of these materials. We suggest that performing EIS measurements with a thin electrolyte layer prepared, for example, by a spray method using a nonreactive solvent and a small amount of binder followed by hydraulic pressing may be one of the convenient measurement methods to avoid the irregularity of thick pellets.

## Experimental Section

### Synthesis of the Li_6_PS_5_Cl Electrolyte

Details of the chloride-doped Li argyrodite synthesis is described in ref (4). A stoichiometric mixture (1:1:1 M) of Li_2_S, LiCl, and β-Li_3_PS_4_ (β-Li_3_PS_4_ was synthesized beforehand using the method described in ref (100)) was dissolved in a small quantity of anhydrous ethanol in Ar atmosphere. This mixture was heated to 90 °C under a vacuum to evaporate the solvent, yielding a white precipitate. This white powder was separated into four batches and further treated for 1 h under vacuum at 200, 300, 400, and 500 °C, respectively, to obtain the final products (Li_6_PS_5_Cl). The final sample colors are shown in Figure S1.

### Preparation of the Li_6_PS_5_Cl Electrolyte Thin Layer^27^

The sample annealed at 500 °C was chosen for the preparation of a thin layer. The slurry composed of 95% Li_6_PS_5_Cl (LPSC) and 5% nitrile butadiene rubber (NBR) in anhydrous ethanol was mixed for several hours. The stainless steel current collector of 10 mm diameter was spray-coated using the H&S Evo Silverline airbrush and compressed argon at 1 bar pressure. The airbrush was equipped with a 400 μm size nozzle to allow the well-homogenized electrolyte particles in the slurry to leave the airbrush nozzle cap and deposit on the support. The thin layer of Li_6_PS_5_Cl@NBR was dried before cell assembly. The 340 MPa pressure was applied to the airtight split coin cell (MTI) to compress the electrolyte layer.

### Structural and Morphological Studies

The phase composition and crystal structure were analyzed using X-ray diffraction (Bruker) with Cu Kα radiation (λ = 1.5418 Å). The Scherrer equation was used to estimate the crystallite size of the obtained materials.^28^ Structural data were obtained through Raman spectroscopy, which was measured using a Renishaw inVia Raman microscope equipped with a 532 nm emission line and a reduced laser power (∼0.1 mW). To determine the morphology of the LPSC powders, pellet and thin film, field emission scanning electron microscopy (Merlin, Zeiss) was used.

### Electrochemical Impedance Spectroscopy (EIS) Measurements

Samples’ ionic conductivities were measured using EIS in the frequency range from 100 mHz to 1 MHz with a 10 mV amplitude using the Solartron SI 1260 impedance analyzer. Densified pellets (10 mm diameter) were made by cold pressing the obtained samples (∼100 mg) under a pressure of 340 MPa (70 bar). The airtight split coin cell (MTI) equipped with stainless steel current collectors as blocking electrodes was utilized. The straight line intercept on the real axis is employed to determine the total ionic conductivity of the material. The temperature-dependent EIS data were recorded from room temperature to 70 °C. The activation energy was calculated based on the Arrhenius plot. In the thin layer LPSC sample, the temperature range was from 0 to 30 °C.

### NMR Relaxation Measurements^7^

Li NMR measurements were carried out using a Varian INOVA 500 spectrometer equipped with a switchable-5 BB VT probe at a magnetic field of 11.74 T and a temperature range from 300 to 400 K. At these conditions, the resonance frequency of lithium-7 is ω_Li_/(2π) = 194.32 MHz. The temperature was calibrated based on the difference in chemical shifts of CH_2_ and OH groups of ethylene glycol.^21^ The longitudinal relaxation time was determined using the inversion recovery pulse sequence with delays from 6.25 ms to 6.4 s in an exponential manner (11 delays in total). Each of the four samples of Li_6_PS_5_Cl heated at 200 °C, 300 °C, 400 °C, and 500 °C was transferred under an inert atmosphere to a separate NMR 1.5 mm outer diameter tube (529-D, Wilmad). Then, each 1.5 mm tube containing the sample was placed in a valved low-pressure NMR tube of 5 mm outer diameter (S-3-500-IPV-7, Norell) and positioned in the center of the tube.

### Quantum Chemical Computations

Energies were computed using the BAND computer program in the Amsterdam Modeling Suite^29−32^ at the level of density-functional theory^33^ with slater-type TZ2P orbitals (triple-ζ with two polarization functions) basis set^34^ and the PBE (Perdew–Burke–Ernzerhof) functional.^35^ The positions of atoms were taken from experimental neutron diffraction data reported in ref (15). Besides phosphorus, crystallographic positions in Li_6_PS_5_Cl are partially occupied; i.e., 41% of lithium atoms are in the Li(1) site and 9% in the Li(2) position; positions in pairs S(2)/Cl(2) and S(3)/Cl(4) are approximately equally occupied. Therefore, we chose a subset of the atomic positions that agrees with the total number of atoms in an elementary cell (Li_24_P_4_S_20_Cl_4_). These atomic positions are listed in Supporting Information Table S2. The periodic conditions were assumed with cubic lattice parameters *a* = *b* = *c* = 9.87 Å. The computations were performed for fixed positions of all atoms except for one lithium atom whose position was varied along the path from one crystallographic lithium position to the other; see Table S4 in the Supporting Information for more details.

## Results

### Morphological and Structural Characteristics of Electrolyte Materials

**Figure 1:**
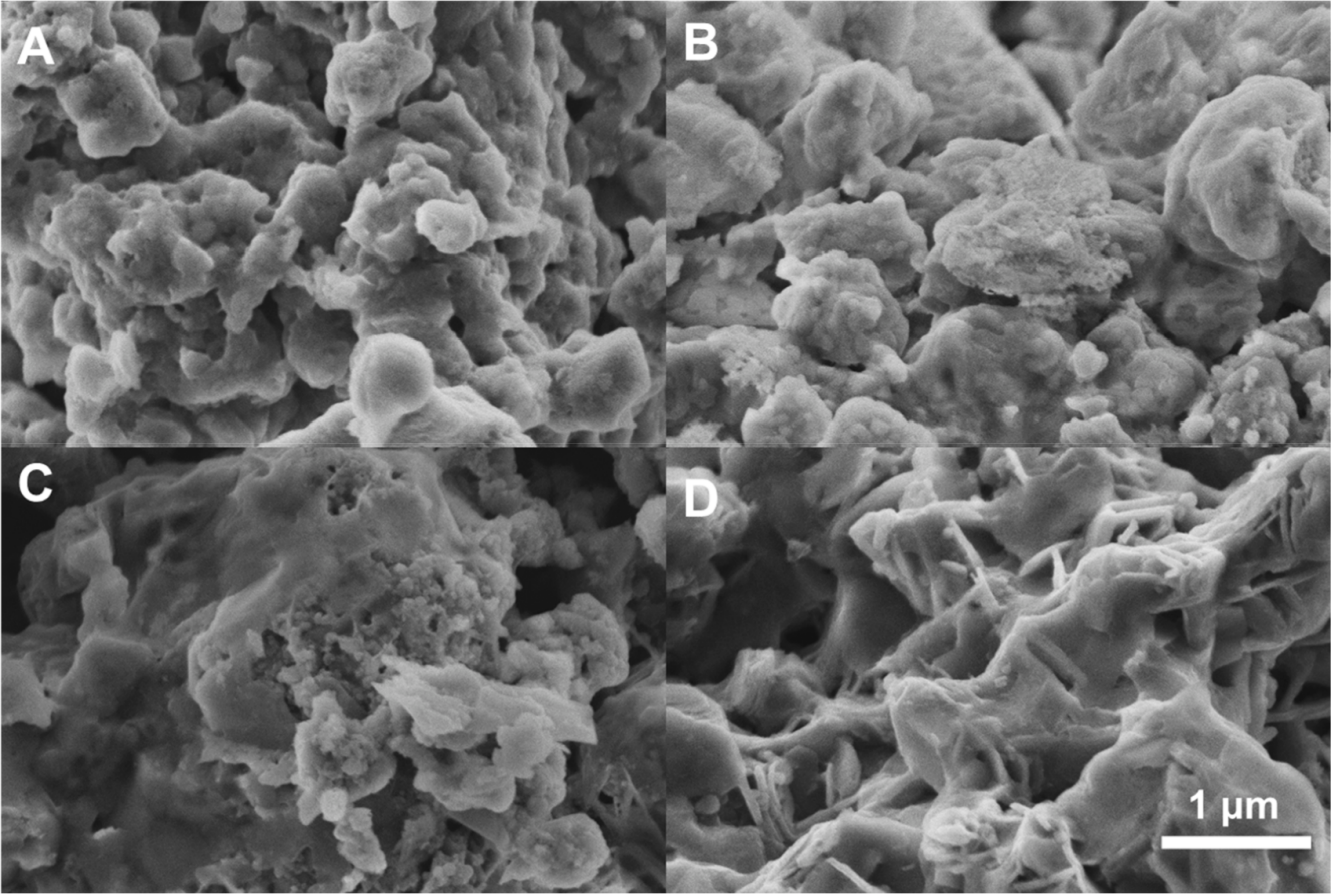
SEM images showing the morphology of samples annealed at (A) 200 °C, (B) 300 °C, (C) 400 °C, and (D) 500 °C.

The obtained samples calcinated at different temperatures (200, 300, 400, and 500 °C) were examined using SEM and XRD techniques. It is noteworthy that the sample color changed from white to brown over vacuum annealing at different temperatures as shown in Figure S1. Significant variations in morphologies of the samples were observed in SEM images (Figure 1). The sample heated at 200 °C demonstrates that this material keeps its postdrying state. Particles are shapeless, grainy, and densely agglomerated. Some visible single grains were about 100 nm or smaller, although the grain boundaries are generally not very visible. This effect is more pronounced in the sample annealed at 300 °C, where surfaces seem more smooth than in the sample annealed at 200 °C. This appearance might suggest high amorphousness. The change in the sample annealed at 400 °C is noteworthy. The previous morphology started to transform into smooth surfaces covering highly interconnected grains. It was an intermediate state between 300 and 500 °C. In the last sample, the particle morphology reminds us of interconnected diversely oriented 100–200 nm thick petals or flakes.

**Figure 2:**
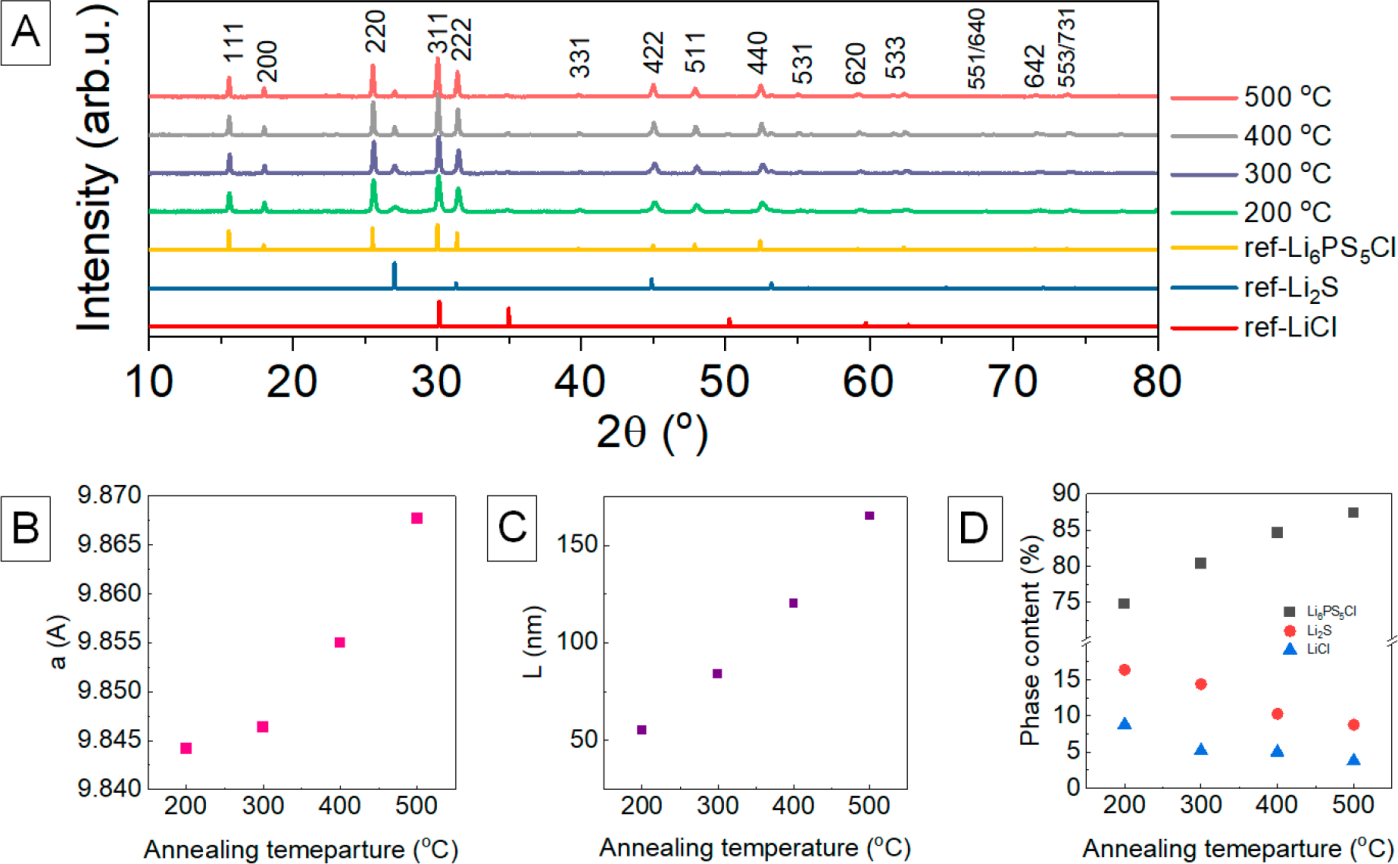
(A) XRD patterns of LPSC samples were annealed at different temperatures. (B) Unit cell parameter calculated using Rietveld refinement. (C) Crystallite size calculated from the Scherrer equation. (D) Phase content.

The diffraction data revealed the crystal nature of the obtained materials (Figure 2A). All diffractograms showed the main phase of Li_6_PS_5_Cl argyrodite that crystallizes in the *F*4̅3*m* space group of the cubic structure.^21^ Raman spectroscopy additionally confirmed the existence of typical Raman lines at 600 (w), 574 (m), 425 (s), 270 (w), and 198 (m) cm^–1^ of Li_6_PS_5_Cl material (Figure S2).^16^ Rietveld refinement analysis was applied to study changes in the crystal structure of lithium argyrodite after annealing at different temperatures. The lattice parameter *a* changes among the samples and increases with annealing temperature (Figure 2B) starting from ∼9.843 Å for samples annealed at 200 °C, followed by a small increase in 300 °C and more significant changes in 400 and 500 °C. The value for the 500 °C sample increased to 9.869 Å, i.e., ∼25 pm expansion in all directions, which gives 0.75% expansion in unit cell volume. The crystallite size also rises with the annealing temperature from ∼60 to ∼170 nm. The samples obtained using a wet-chemical method contain additional phases: LiCl and Li_2_S, both crystallizing in the same *Fm*3̅*m* space group. Minor reflexes at 22.23 and 23.17°, probably originating from Li_3_PO_4,_ were also detected. The total amount of impurities was reduced once the sample was annealed at 500 °C. These are typical impurities spotted in halide-doped lithium argyrodite materials.^4,17−19,36^ The XRD studies showed that samples prepared in higher quantities (∼3 g) contain more postreaction impurities than samples previously prepared in small quantities (∼500 mg ^4^).

### Ionic Conduction and Activation Energy Studied by EIS Technique

The ionic conductivity of LPSC samples was measured using the EIS technique. As expected, the sample prepared at the lowest temperature showed the lowest conductivity of about 2.87 × 10^–5^ S cm^–1^ at room temperature. Heating samples under a vacuum at higher temperatures improved total ionic conductivity; i.e., samples annealed at 300, 400, and 500 °C have ionic conductivities at room temperature of 0.296, 0.549, and 0.793 mS·cm^–1^, respectively. The LPSC sample annealed at 500 °C prepared as a thin layer showed an ionic conductivity of 0.743 mS·cm^–1^ at room temperature, which agrees with the results obtained for a pellet.

**Figure 3:**
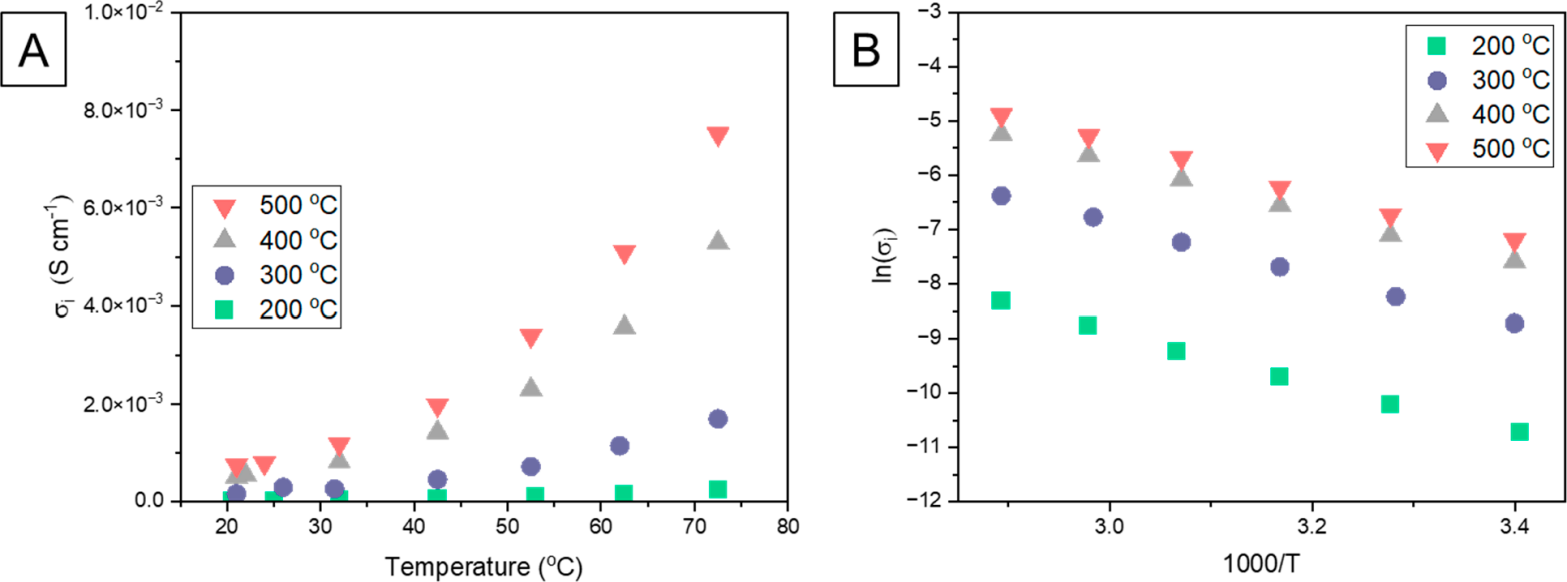
Temperature dependence of the ionic conductivity of annealed LPSC samples obtained from EIS (A) and its Arrhenius plot (B).

The temperature-dependent EIS was applied to investigate the activation energy of Li-ion conduction. Figure 3A shows how the total ionic conductivity changes over the heating of samples between 20 and 70 °C. Figure 3B represents Arrhenius plots of ionic conductivities vs temperature for LPSC annealed at different conditions. According to eq 2, all samples show linear relations, with the slopes proportional to the activation energy *E*_a_. Noticeably, all slopes are similar and correspond to the activation energy of ∼0.4 eV (∼39 kJ mol^–1^) in good agreement with our previous work^4^ and other literature data.^10,11^ It was shown that a slight decrease of energy *E*_a_ is observed for the 500 °C sample (Figure S3), which might be related to a lower impurity content, higher crystallinity, and bigger unit cell parameters.

**Figure 4:**
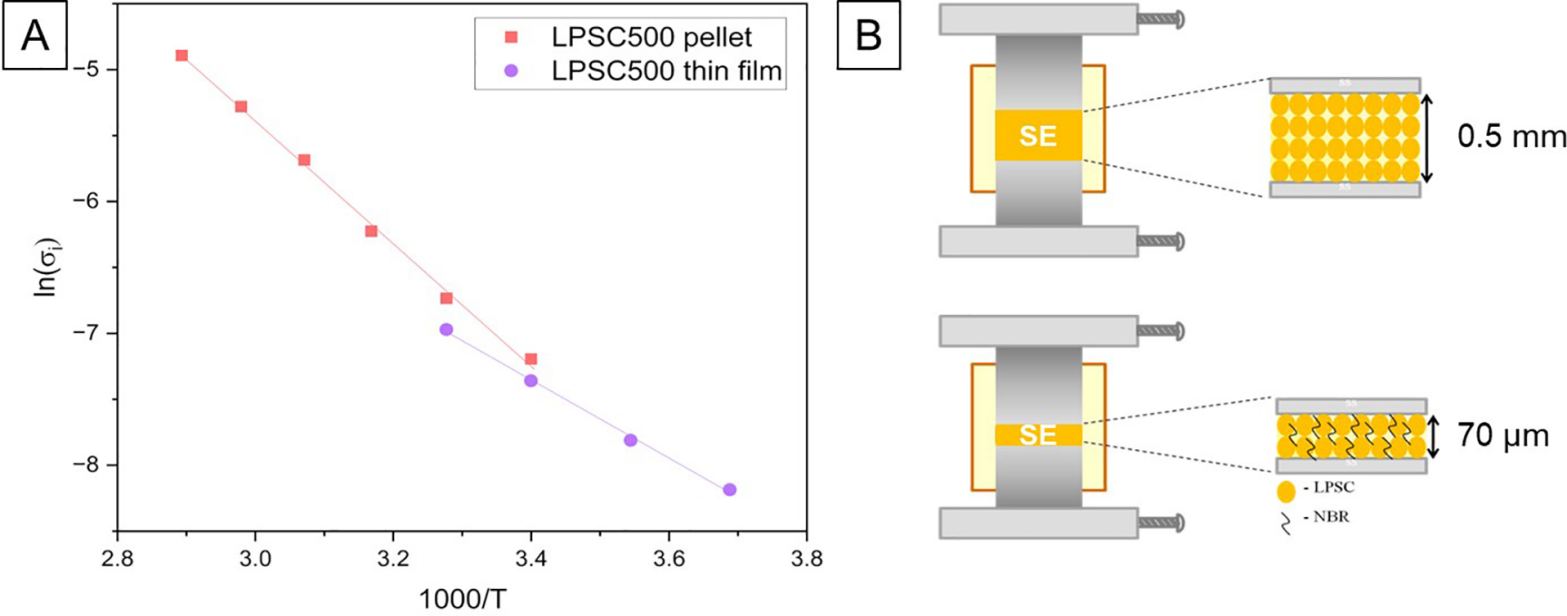
Comparison of Arrhenius plots obtained from (A) the pellet and (B) the thin film EIS setup measured on of LPSC sample annealed at 500 °C.

Figure 4 compares the Arrhenius plot of a bulk LPSC material pellet (0.6 mm thickness) and an LPSC thin layer (about 70 μm thickness). The thin layer LPSC500 sample prepared by the spraying method showed a different slope angle than the pellet prepared from the material annealed at 500 °C. Surprisingly, the total activation energy calculated from eq 2 reveals the *E*_a_ value of 0.26 eV (24.69 kJ mol^–1^), almost 2 times lower than one obtained through the commonly used pellet setup.

**Figure 5:**
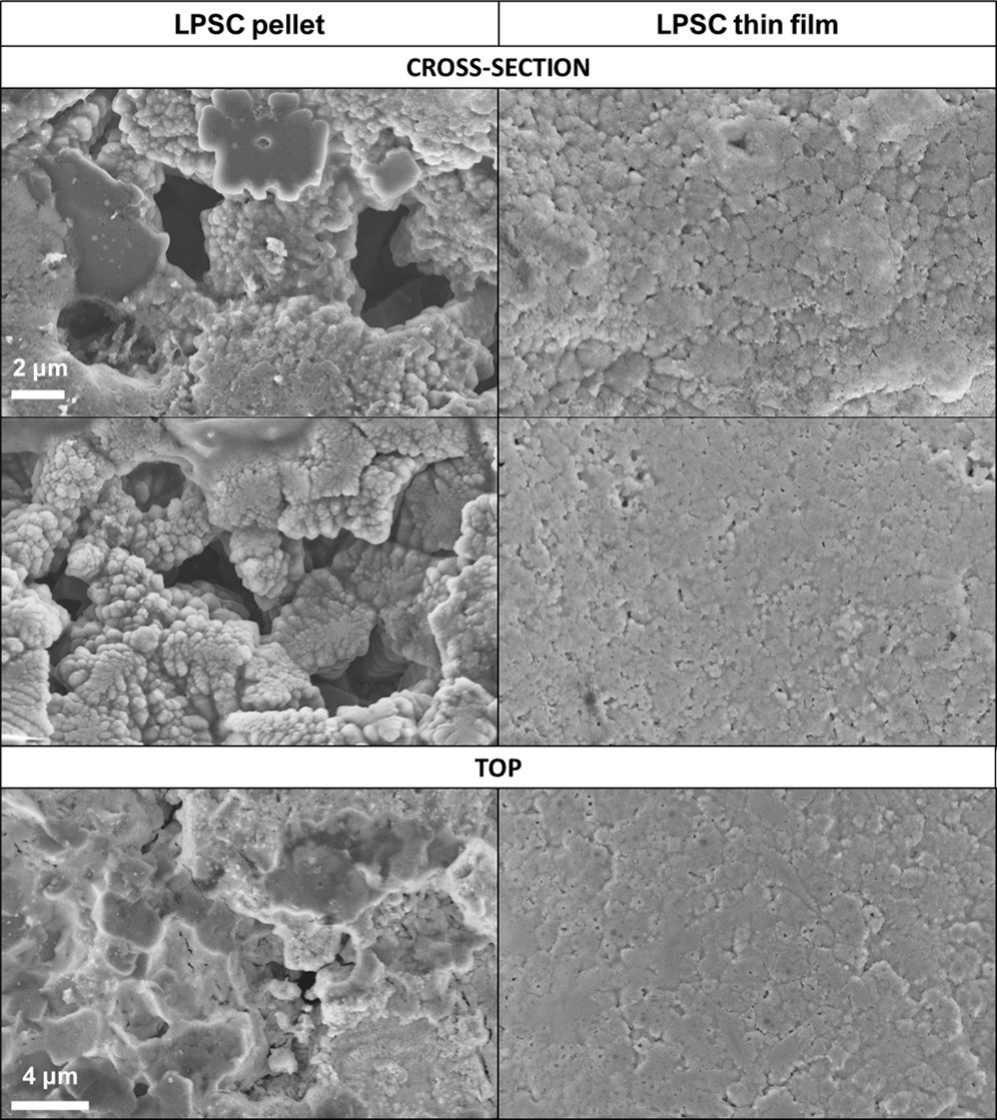
Comparison of SEM images showing morphology of LPSC sample annealed at 500 °C within thick pellet and thin film showing a porosity of cross sections and top surfaces Arrhenius plots obtained from (A) the pellet and (B) the thin film EIS setup measured on of LPSC sample annealed at 500 °C.

Based on recent finding of Liu et al.^20^ and our results, we see that the activation energy values depend on porosity of the sample, which is highly dependent on its preparation method. Figure 5 shows the comparison of the cross-section and top surfaces of the pellet and thin film samples. When preparing a research-standard pellet (10 mm in diameter from ∼80–100 mg of LPSC powder), the formation of numerous of voids, cavities, and tunnels within its structure can be observed due to powder compaction issues. The pellet top surface that has a physical contact with a current collector also shows a number of voids which might additionally disturb the electrical response since the real contact area will be smaller than in eq 1. The SEM histogram analysis revealed a pellet porosity of an average ∼11%, which is in good agreement with its relative density of 90.3%.

This macroporosity caused by particle misalignment will not only lower the contact area with the current collector but also elongate the diffusion pathway within the pellet. In the case of thin film sample, the layer and its top surface are significantly more condensed and compact. There are tiny voids (in average 2.5%), but generally particles and grains are closely packed and the estimated density of the thin film layer was about 97.5% compared to ∼89% of the pellet sample. Each particle and furthermore grain seem to be interconnected through thin grain boundaries in a progressive way, making the entire electrolyte layer more integrated and close to an ideal (though polycrystalline) bulk sample. As shown schematically (Figure S4), the ionic pathway will be affected by those macroscopic gaps within the pellet sample. Some particles or grains might be isolated or might have a minor physical contact between particles or grains and thus additionally disturb the EIS response. Liu et al. obtained the activation energy of 0.3 and 0.33 eV for samples with densities of 95.37 and 92.76%.^20^Figure S5 shows the clear porosity dependence on obtained activation energy values when comparing both experimental result sets. With an increased porosity, the activation energy increases. That is why the sample preparation and keeping the porosity as low as possible might be crucial for obtaining low energy barriers within layer. The effect of the morphology and porosity should be further studied. It is recommended that crystal size, particle size, and sample porosity will be included in future works in the field to thoroughly investigate the relationship between activation energy, ionic conductivity, and these morphological parameters.

### Ionic Conduction and Activation Energy Studied by NMR Spectroscopy

**Figure 6:**
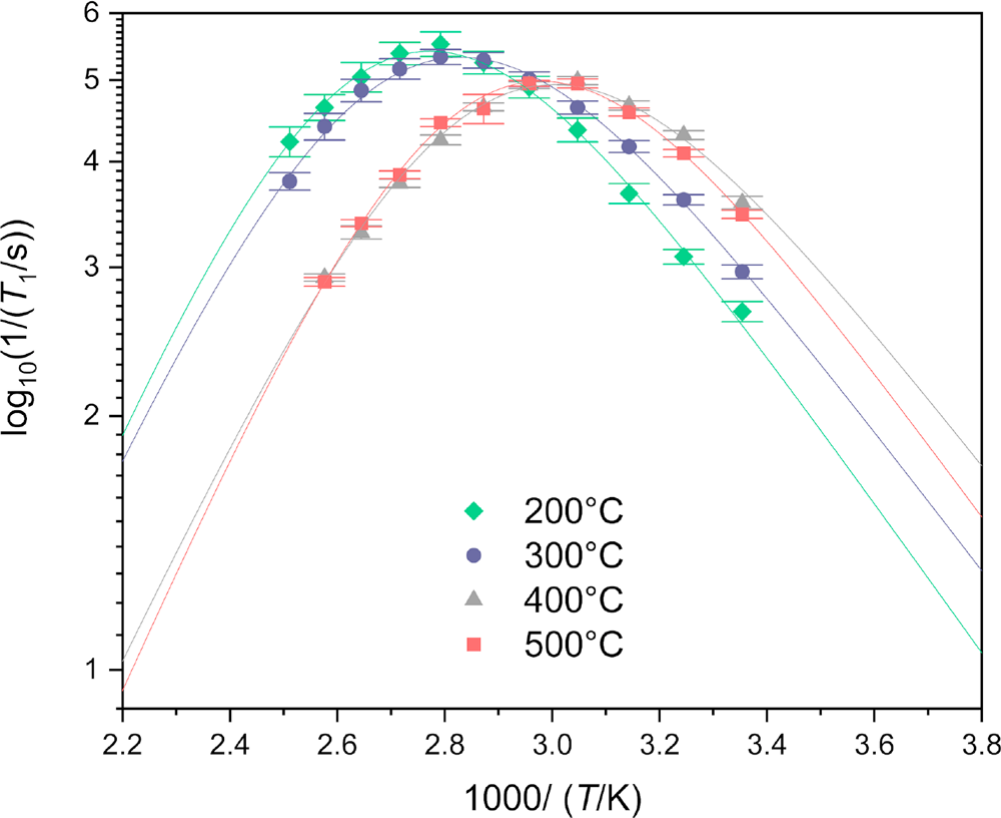
Temperature dependence of the longitudinal relaxation rate (*T*_1_^–1^) of Li_6_PS_5_Cl (points). The curves are the best fits of the function  to the experimental data, where  and β = 1.6. The coefficients *A*, *B*, and *C* for each sample are given in Supporting Information Table S4.

The ^7^Li NMR spectrum of Li_6_PS_5_Cl consists of a single broad line (fwhm ∼0.7 kHz) at the chemical shift of ∼2.8 ppm relative to the 1 M lithium chloride D_2_O solution. The peaks of lithium occupying different crystallographic sites, i.e., Li(1) and Li(2), were unresolved under the conditions of the conducted measurements. To further understand lithium conductivity in our samples, temperature-dependent lithium-7 relaxation measurements were conducted. In order to estimate lithium atom mobility in microcrystals of Li_6_PS_5_Cl, we measured the longitudinal ^7^Li relaxation time in a 100 °C span of temperatures (from 25 °C to 125 °C). The measured values of *T*_1_ are shown against a reciprocal of the temperature in Figure 6 and listed in Table S4 of the Supporting Information.

According to the Bloembergen–Purcell–Pound theory,^37^ the *T*_1_ relaxation time depends on the modulation of the strength of lithium magnetic moment interactions that changes its energy. In a Li_6_PS_5_Cl crystal, the lithium atom energy is affected by the interaction between the lithium quadrupole moment and an internal electric field gradient, dipolar interactions between magnetic moments of lithium atoms, and the anisotropy of the nuclear magnetic shielding tensor. In general, each of these interactions causes relaxation that depends differently on the lithium spin precession frequency and the diffusion of lithium through the Li_6_PS_5_Cl crystal lattice. To make the problem tractable, we assumed that the variation of the *T*_1_ relaxation time shown in Figure 6 follows the Lorentzian-like spectral density function, i.e.,
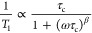
2awhere ω_Li_ is the spin precession frequency equal to 194.32 MHz at the magnetic field strength of 11.74 T, and parameter β phenomenologically describes the deviation from the exponentially decaying autocorrelation function. In particular, the plot of 1/*T*_1_ in the function of 1/*T* is symmetric if β = 2.^11^ The time τ_c_ is the autocorrelation time of lithium atoms that fulfills the Arrhenius law:
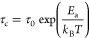
3In eq 3, *E*_a_ is the average activation energy of the lithium atom; *k*_B_*T*/*e* = 25.4 meV at *T* = 298 K. The assumption of the Lorentzian-like spectral density function may be justified by noting that the most efficient relaxation occurs when the time scale of jumps of the lithium atom between crystallographic positions coincides with the spin precession frequency (ω ≈ τ_c_). The dependence of the relaxation rate 1/*T*_1_ given by eqs 2 and 3 fits well to the collected data (see Figure 6). In our case, β turned out to be 1.6 for all samples, which is in good agreement with previous data measured in the higher range of temperatures.^11^

The obtained data indicate two different activation energies (*E*_a_) calculated from the left and right curve arms using the obtained slope and β parameter. The activation energy from the low-temperature side is explained by short-range or localized ion dynamics in double-well potentials, including also highly correlated forward–backward Li jumps (e.g., intracage jump processes).^11,38^ The high-temperature side can describe the activation energy coming from many jump processes that occur during one Larmor precession of the spin (generally described as long-range diffusion).^11^ The activation energy of the short-range Li dynamics is ∼170 meV at *T* = 298 K, and it is almost independent of the heating temperature of the samples. A comparable pattern was noted in the context of long-range diffusion, wherein the activation energy was found to be approximately 280 meV. Noticeably, both values of the activation energy types are smaller than those calculated from EIS experiments for pelletized samples, showing that the additional process, such as grain boundary resistance and the resistance at the physical contact between particles, limits the lithium diffusion within the thick pellet. It is pronounced that the value of activation energy of the thin layer corresponds very well to the long-range diffusion calculated from NMR measurements, showing that the sample preparation method has a huge impact on the obtained results and interpretation.

A more pronounced effect of sample heating was observed for the autocorrelation time τ_c_ for both (long and short) Li diffusion distance ranges. Both values showed a decreasing trend from (51.39 ± 4.72) ns to (27.35 ± 1.16) ns for long-range diffusion and from (0.522 ± 0.018) ns to (0.303 ± 0.004) ns for short-range lithium jumps after annealing samples at 200 and 500 °C, respectively (Figure S6). Assuming that one can identify the autocorrelation time τ_c_ with the hopping time between crystallographic positions of lithium in a Li_6_PS_5_Cl crystal and combining the Einstein–Smoluchowski equation with the Nernst–Einstein equation,^39^ the ionic conductivity σ_Li_ is
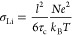
4where *l* is the distance between lithium positions (2.0 Å), *N* = 2.5 × 10^28^ m^–3^ is the density of lithium, and *e* = 1.602 176 634 × 10^–19^ C is the elementary electric charge.

Based on that assumption, the ionic conductivity corresponding to the short-range lithium dynamics estimated for LPSC crystal from NMR measurement (σ_NMR_) increases with increasing sample calcination temperature and, in general, is in a range of tens of mS cm^–1^, which is in good agreement with values obtained in the literature for same class of materials.^40^ It is also noticeable that the total ionic conductivity of pellets and our thin layer sample is much lower than the σ_NMR_. This confirms that the conduction of lithium ions is limited through the long-distance lithium diffusion within the grain and grain boundries.

### Calculations of the Energy Change across Li Jumps

In addition to the experimental data, quantum chemical computations using density-functional theory (DFT) were performed. The crystal lattice and positions of atoms chosen for this calculation were established using the crystal structure refined using neutron diffraction reported in ref (15). Lithium hopping within the crystal lattice involves the movement of Li ions from one site to another within the lattice structure. There are two types of lithium ion positions (Li(1) and Li(2)), both localized at 48h Wyckoff sites. Only 50% of sites are occupied by Li ions, and Li(1) types occupy 41%, whereas Li(2) occupy 9% of available sites. Intracage Li jumps refer to the Li ion movement within a tetrahedral Li cage formed around the S or Cl located at the 4d Wycoff position. Each cage consists of four Li site hexagons. There are six Li ions within one cage, which statistically gives one or two Li^+^ occupations per hexagon. Intercage hopping involves the Li ion movement between different Li cages connecting two Li(1)–Li(1)* positions or Li(2)–Li(2)* positions. Knowing that, we randomized the occupancy of Li within the structure, taking into account the occupancy probability, and picked one mobile Li^+^ to simplify the DFT calculations. We also kept the randomized S and Cl (50%:50% occupancy) positions in the lattice.

**Figure 7:**
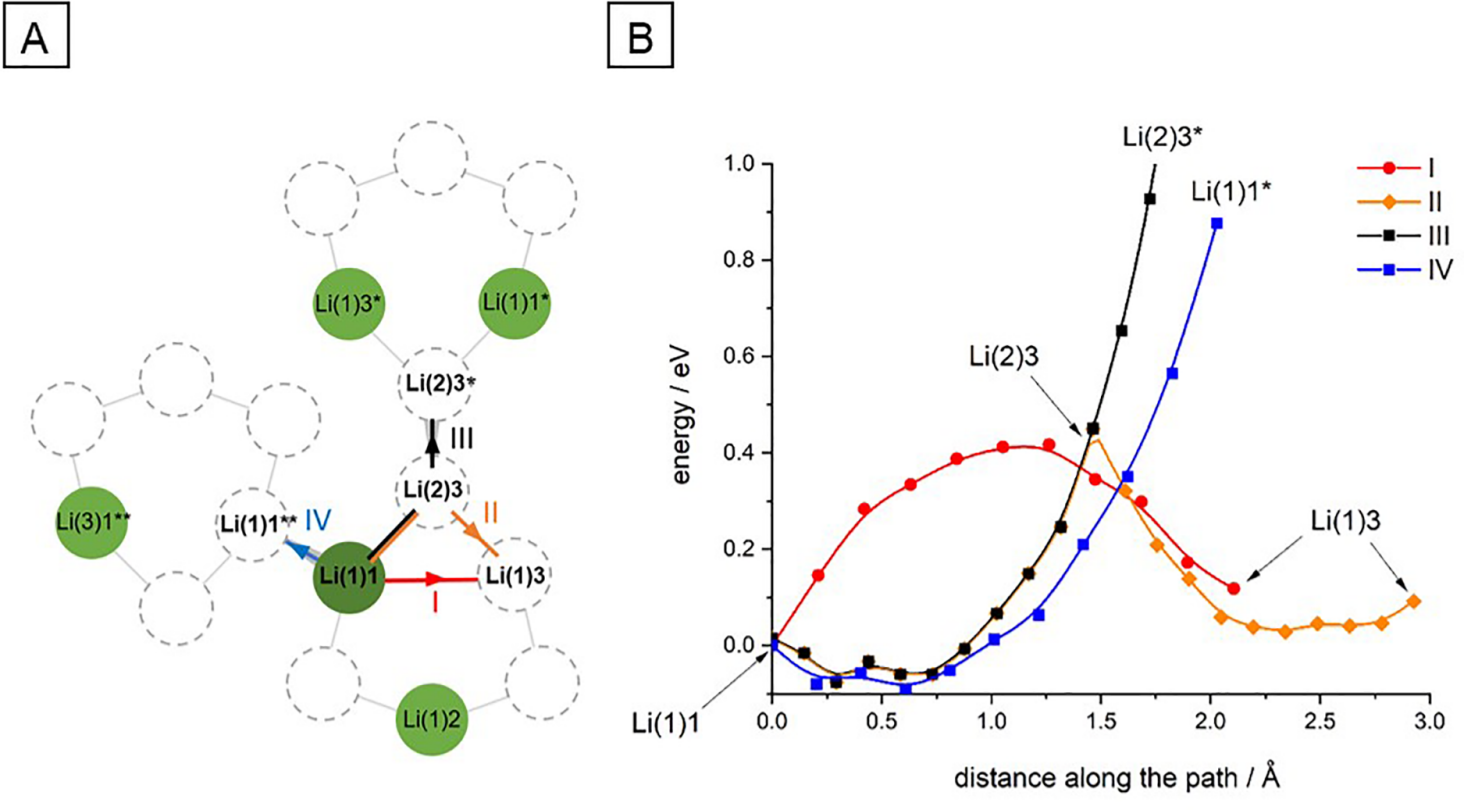
(A) Diffusion paths of a lithium ion (dark green) which moves from the position Li(1)1 to the Li(1)3 position directly (path I; the red arrow) and through intermediate position Li(2)3 (path II; the orange arrow) to the position Li(2)3* (path III, the black arrow) and to the other adjacent cluster to the position Li(1)1** (path IV, the blue arrow). The green cycles represent occupied positions by lithium atoms, while the dash-line circles those that are unoccupied. (B) Energy of the crystal along the path the lithium atom moves. Path III is shown only up to 1 eV; the energy for the lithium atom position Li(1)** is about 10 eV. Points are computed energies, and lines are guides for the eye.

Both intracage and intercage jumps are important for ionic conduction and contribute to the overall Li ion mobility within the solid-state electrolyte. We tracked the changes in energy of the Li ion traveling within the LPSC crystal lattice. The selected pathways are shown in Figure 7A. One can see that depending on which pathway we chose, the energies of Li jumps are differently affected (Figure 7B). A close distance Li(1)1–Li(2)3 path within a hexagon (1.468 Å) consumes the least energy in decimal values of eV. A further travel to the equivalent position Li(1)3 decreases the energy, making this two-step diffusion most probable. A longer distance intracage jump between two equivalent Li(1) positions (Li(1)1–Li(1)3) gives a rise in energy over the entire distance of 2.113 Å, making it less favorable. The next two chosen paths were more problematic. In the case of intracage Li(1)–Li(1)** jump (2.035 Å) between two hexagons, the energy is highly increased. Similarly, with an even higher effect, the intercage jump between Li(2)3 and Li(2)3* greatly raised the energy and made such movement unlikely. Since our model assumed a rigid crystal structure with one mobile Li ion, the stiff Li ions in the simulated lattice possibly disturbed the final calculated values. Since the Li^+^ movement within one hexagon seems to consume a minor amount of energy, intra- or intercage jumps between hexagons should involve low-energy Li(1)–Li(2) and Li(2)–Li(1) jumps and overall Li position rearrangements. More sophisticated calculations would have to be employed to simulate the synchronized movement of all Li ions in the LPSC lattice, although this calculation indicates that a single Li^+^ movement causing an increase of Li ion numbers within an individual hexagon could potentially impact the Li ionic conductivity. This is because it will require the displacement of pre-existing Li ions within that hexagon if such movements are even possible due to the interaction of the adjacent hexagons and their Li ion positions. This mechanism might limit the diffusion within the crystal.

## Discussion

**Figure 8:**
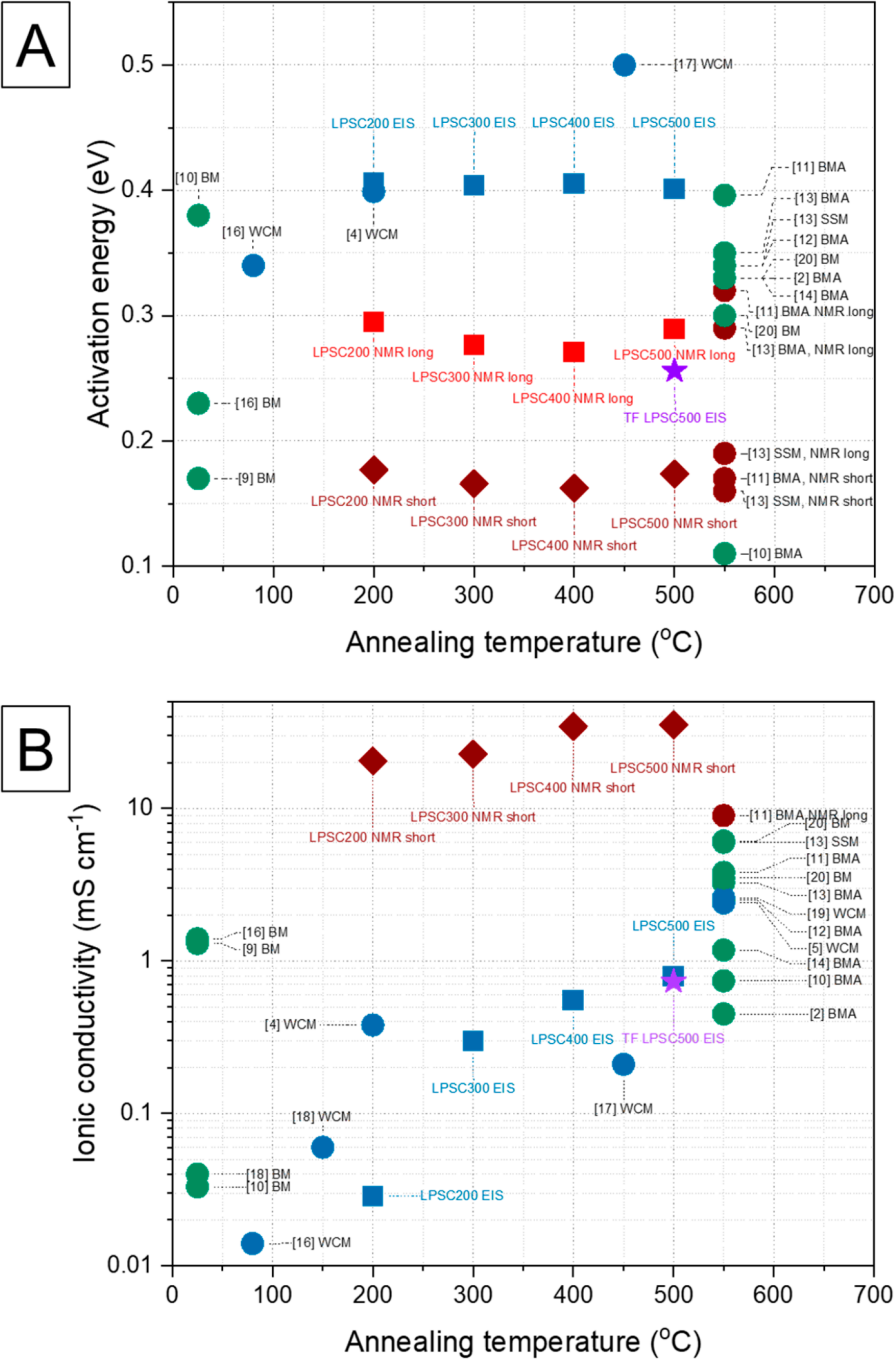
Comparison of the literature data and our experimental data in terms of (A) activation energy and (B) Li ionic conductivity in correlation with sample annealing temperature. BM, ball milling; BMA, ball milling + annealing; WCM, wet chemical method; SSM, solid-state method.

Our findings on the activation energy and Li ionic conductivity of short-range and long-range diffusion were compared with our EIS results and the literature data (Figure 8).^2,4,5,9−20^ We observed that the activation energy of short-range Li diffusion was about 170 meV at *T* = 298 K and was almost independent of the heating temperature of the samples. Those values were much smaller than those calculated from EIS experiments for pelletized samples and very similar to the previous estimates from the NMR meaesurements.^11,13^ This significant difference in comparison to EIS estimated values of total activation energy of Li diffusion indicates that additional effects, such as grain boundary resistance and the resistance at the physical contact between particles, have a significant contribution to the limitation of Li diffusion within the thick pellet. The activation energy of long-range diffusion was found to be approximately 280 meV, which is comparable to the literature data on solid-state NMR. It is worth noting that the value of the activation energy of our thin layer sample corresponds well to the long-range diffusion calculated from NMR, showing that the sample preparation method had a significant impact on the obtained results and interpretation. Our samples synthesized using the wet chemical method have higher activation energies than those synthesized using the ball-milling method followed by prolonged annealing at 550 °C, suggesting that the morphology of the sample and/or crystal quality might impact those characteristics.

Our results for the total Li ionic conductivity were consistent with the literature data. Total ionic conductivity increases with annealing temperature and is much lower than the σ_NMR_, confirming that the conduction of lithium ions is limited through the long-distance lithium diffusion within the grain and grain boundries. The material preparation using the wet chemical methods reported in the literature is generally finalized with lower temperature heat treatment, which causes slowing the Li mobility within the crystal. Our systematic study shows how crystallization and sample optimization can improve crucial material parameters. We also suggest that the total ionic conductivity is 2 orders of magnitude smaller than that of short-range. Thus, we assume that the Li mobility is affected over the long-range diffusion which is reflected in the autocorrelation time τ_c_ for both (long and short) Li diffusion distance ranges and corresponding activation energies. Those long-range limitation factors such as variations in energy barriers along the Li^+^ pathways within crystal (Figure 8A), crystal disorder or defects, grain boundaries, particles’ physical contact, etc. might significantly lower the ionic conductivity within the macroscopic sample.

Our novel thin-layer deposition method gives very attractive results for applying solid electrolyte layer preparations for EIS measurements and battery assembly. The thin layer sample fits well into the long-range Li diffusion activation energy value calculated from the NMR experiment. This finding suggests that the thin-layer sample has a smaller impact on grain boundaries and less physical particle contact resistance thanks to the application of micrometer thickness.

Unfortunately, numerous research papers on Li argyrodites lack a comprehensive analysis of the crystal size from diffraction data, as well as particle size determined from microscopic or other methods, even though information on the grain/crystal size and particle size of LPSC is very important to unravel the intricate nature of ionic conduction within the crystal, interphases, and grain boundaries. Our findings indicate a clear correlation between the expansion of the *a* unit cell parameter and the increase in Li ionic conductivity in LPSC materials. This observation aligns well with the systematic research conducted by Yubuchi et al. on Li_6_PS_5_Br material.^41^ Surprisingly, our observations do not exhibit a similar correlation when compared to the literature data reported for LPSC (Figure S7). While some experimental values align with this trend, others appear to deviate randomly. What is more surprising, there is an evident lack of literature data on crystal size of LPSC; we found only four additional research findings to compare with our data (Figure S8). There seems to be a potential correlation between Li ionic conductivity and LPSC crystal size, analogous to Li_6_PS_5_Br material, where achieving high conductivities relied on controlling the ratio of amorphous and crystalline phases.^41^ For the LPSC, the highest ionic conductivity (σ_ion_) has been observed in the case of the largest crystal size reported. This observation suggests that the conduction mechanism in these materials is primarily limited by grain boundaries and interfaces, which corresponds closely to our study and the results obtained from the thin layer sample.

## Conclusions

This study presents a comprehensive investigation into the activation energy and ionic conductivity of lithium argyrodite materials using electrochemical impedance spectroscopy and temperature-dependent solid-state nuclear magnetic resonance spectroscopy. Specifically, Li_6_PS_5_Cl material synthesized via the wet-chemical method and annealed at different temperatures was selected for this study. The best performed sample was furthermore prepared using the new thin-layer deposition method to obtain only a 70 μm thick electrolyte layer. In addition to EIS and NMR analyses, structural and morphological data were used to correlate the conduction and activation energies of these materials. The asymmetry of the Arrhenius plots of the ^7^Li spin–lattice relaxation rates observed in this study was attributed to various Li-ion diffusion processes occurring at different length scales, which are hardly obtained in the EIS experiments. The obtained data suggest the presence of two different activation energies related to short- and long-range Li diffusion. Values of *E*_a_^long^, which are related to the long-range lithium diffusion, correlate well with the total activation energy measured on the thin layer sample using the EIS method. The activation energy for short-range Li dynamics is about ∼170 meV at *T* = 298 K, while the activation energy for long-range diffusion is around 280 meV. Both values are lower than the one calculated from EIS experiments for the thick pellets, indicating that additional processes, such as grain boundary resistance and physical contact resistance, limit lithium diffusion. This study also highlights the importance of sample preparation methods in obtaining more precise results. Our findings demonstrate a correlation between increased Li ionic conductivity and the expanded *a* unit cell parameter in LPSC materials, consistent with prior research on Li_6_PS_5_Br. Remarkably, higher ionic conductivity is observed in LPSC with larger crystal size, indicating the limiting role of grain boundaries and interfaces in Li ion conduction, in line with our thin layer sample results. Understanding the conduction mechanisms and activation energies involved in Li hopping is crucial for the development of high-performance solid-state batteries. Thus, the observed trend of increasing activation energy with increasing porosity highlights the critical importance of precise sample preparation techniques to minimize porosity and achieve reduced energy barriers in the layer. Therefore, a comprehensive study of the effects of morphology and porosity is still needed. Accordingly, it is recommended that future research in this field consider crystal size, particle size, and sample porosity as fundamental parameters to accurately explain the complex relationship between activation energy, ionic conductivity, and these structural and morphological features. Overall, our study provided insights into the fundamental mechanisms governing Li ion transport in solid-state batteries and established a reliable framework for further investigations in this area.

## Supporting Information Available

Detailed information about literature data on experimental total ionic conductivity at room temperature (298 K) and calculated activation energy of Li_6_PS_5_Cl material; coordinates of atoms used in quantum chemical computations; diffusion paths of the lithium atom no. 21; longitudinal relaxation time of 7Li in seconds measured from 25 to 125 °C for samples prepared at temperatures 200, 300, 400, and 500 °C; coefficients *A*, *B*, and *C* of the best fit to the experimental data; picture of LPSC samples annealed at different temperatures; Raman spectra of all LPSC samples annealed at 200, 300, 400, and 500 °C; activation energies calculated from the temperature-dependent EIS measurements for all LPSC samples annealed at 200, 300, 400, and 500 °C; schematic of Li^+^ ion conduction pathways; experimentally calculated activation energies in relation to relative density of solid electrolyte sample; autocorrelation time (*τ*_c_) of lithium atoms that fulfills the Arrhenius law calculated for both arms (short- and long-range) of the temperature-dependence of the longitudinal relaxation rate (*T*_1_^–1^) function; comparison of the literature data and our experimental data in terms of the unit cell parameter *a* in correlation with Li ionic conductivity; comparison of the literature data and our experimental data in terms of crystal size in correlation with Li ionic conductivity (PDF)
