# Supplementary material for: Lithium Transport Studies on Chloride-Doped Argyrodites as Electrolytes for Solid-State Batteries
Source: ACS Appl Mater Interfaces. 2023 Nov 3;15(46):53417–28. doi: 10.1021/acsami.3c10857 (PMC10685348; doi:10.1021/acsami.3c10857)
Supplement: Supplementary file 1 — am3c10857_si_001.pdf [file am3c10857_si_001.pdf]

Supporting Information

Lithium Transport Studies on Chloride-doped Argyrodites as Electrolytes for Solid-State Batteries

Dominika A. Buchberger[1,], Piotr Garbacz[1,*], Krzysztof Słupczyński[1], Artur Brzezicki[1,2], Maciej Boczar[1], Andrzej Czerwiński [1,*]*

[1] Faculty of Chemistry, University of Warsaw, Pasteura 1, 02-093 Warsaw, Poland

[2] Adamed Pharma SA, Pieńków, Poland

Corresponding Authors

* Dominika A. Buchberger

Faculty of Chemistry, University of Warsaw, Pasteura 1, 02-093 Warsaw, Poland

e-mail: d.buchberger@uw.edu.pl

* Piotr Garbacz

Faculty of Chemistry, University of Warsaw, Pasteura 1, 02-093 Warsaw, Poland

e-mail: pgarbacz@uw.edu.pl; tel: +48-22-55-26-346

Supporting material – table of contents

Table S1. The experimental total ionic conductivity at room temperature (298 K) and calculated activation energy of Li_6PS_5Cl material. 3

Table S2. Coordinates of atoms used in quantum chemical computations 6

Table S3. Diffusion paths of the lithium atom no. 21 listed in Tab. S1 (coordinates in Å). 7

Table S4. Longitudinal relaxation time of ^7Li in seconds measured in temperatures from 25 to 125 °C for samples prepared at temperatures 200, 300, 400, and 500 °C 8

Table S5. The coefficients A, B, and C of the best fit of the function $f(x) = \frac{Ae^{Bx}}{1 + Ce^{\beta Bx}}$ to the experimental data given in Tab. S3. 8

Figure S1. Picture of LPSC samples annealed at different temperatures. 9

Figure S2. Raman spectra of all LPSC samples annealed at 200, 300, 400, and 500 °C. 9

Figure S3. Activation energies calculated from the temperature-dependent EIS measurements for all LPSC samples annealed at 200, 300, 400, and 500 °C. 10

Figure S4. Schematic of Li^+ ion conduction pathways. 11

Figure S5. Experimentally calculated activation energies in relation to relative density of solid electrolyte sample. 12

Figure S6. The autocorrelation time (τ_c) of lithium atoms that fulfills the Arrhenius law calculated for both arms (short- and long-range) of the the temperature-dependence of the longitudinal relaxation rate (T_1^{-1}) function. 12

Figure S7. The comparison of the literature data and our experimental data in terms of *a* unit cell parameter in correlation with Li ionic conductivity. BM- ball milling, BMA – ball milling + annealing, WCM – wet chemical method, SSM – solid-state method. 13

Figure S8. The comparison of the literature data and our experimental data in terms of crystal size in correlation with Li ionic conductivity. BM- ball milling, BMA – ball milling + annealing, WCM – wet chemical method, SSM – solid-state method. 13

Table S1. The experimental total ionic conductivity at room temperature (298 K) and calculated activation energy of Li_6PS_5Cl material.

Ref.	Material preparation method	Ionic conductivity measurements	Total ionic conductivity (mS cm^{-1})	Activation energy (eV)
Solid-state methods				
[2]	Mechanical milling: Li_2S, P_2S_5, LiCl; 600 rpm, 20 h; 5 h at 550 °C	EIS, 10 mHz to 200 kHz<br>- SE pellets: φ 10 mm; ~1.5 mm thickness<br>- C as blocking electrodes	0.45	0.33
[9]	Mechanical milling: Li_2S, P_2S_5, LiCl; mortar 15 min, 600 rpm, 20 h, no annealing	EIS, 10 Hz to 1 MHz<br>- SE pellets: φ 10 mm; ~1 mm thickness<br>- C as blocking electrodes	1.3	0.17
[10]	Mechanical milling: Li_2S, P_2S_5, LiCl; 600 rpm, 20 h; annealed 5 h at 550 °C	EIS, 1 Hz to 10 MHz<br>- SE pellets: φ 10 mm; ~1.5 mm thickness<br>- SS as blocking electrodes	0.033 (BM)<br>0.74 (BMA)	0.38<br>0.11
[11]	Mechanical milling: Li_2S, P_2S_5, LiCl; 400 rpm, 4 h; annealed 7 days at 550 °C	EIS, 10 mHz to 10 MHz<br>- SE pellets: φ 5 mm<br>- Au as blocking electrodes<br>NMR, ^7Li, 7 T, temp. range: −100 to +160 °C, 116 MHz	3.8<br>9.0	0.396<br>0.17<br>0.32
[12]	Mechanical milling: Li_2S, P_2S_5, LiCl; 380 rpm, 17 h; pelletized and annealed 5-7 h at 550 °C	EIS, 100 mHz to 1 MHz<br>- SE pellets: φ 10 mm; 0.63 mm<br>- In as blocking electrodes	2.5	0.34
[13]	(SSM) Mechanical milling: Li_2S, P_2S_5, LiCl; 110 rpm, 1 h; annealed 10 h at 550 °C	EIS, 1 Hz to 1 MHz, amp. 10 mV<br>- SE pellets: φ 10 mm; ~3 mm, 8 tons<br>- SS as blocking electrodes	5.99 (SSM)	0.34 (SSM, EIS)<br>0.16 (NMR_{local})<br>0.19 (NMR_{long})

	(BM) Mechanical milling: Li_2S, P_2S_5, LiCl; 550 rpm, 16 h; annealed 5 h at 550 °C	ssNMR, ^7Li, 155.506 MHz, ref. 0.1 M LiCl, temp. range: −100 to +180 °C	3.25 (BMA)	0.35 (BMA, EIS)<br>0.09 (NMR_{local})<br>0.29 (NMR_{long})
[14]	Mechanical milling: Li_2S, P_2S_5, LiCl; 550 rpm, 16 h; annealed 5 h at 550 °C	ssNMR, ^7Li, 155.506 MHz, temp. range: −120 to +180 °C	1.18	0.33 (NMR_{bulk})
[15]	purchased from NEI Corporation company	EIS, 100 mHz to 1 MHz, amp. 10 mV<br>- SE pellets: φ 10 mm; ~0.75 mm, 4.3 tons<br>- SS as blocking electrodes<br>Temperature-dependent static ^7Li NMR, 77.8 MHz, quadrupolar-echo pulse sequence	3.4<br>3.9 (NMR)	0.28<br>0.14 (NMR_{local})<br>0.27 (NMR_{long})
[20]	Mechanical milling: Li_2S, P_2S_5, LiCl;<br>(1) powder sample: annealed 4h at 550 °C then was pressed into pellets<br>(2) pellet sample: LPSC pelletized annealed at 550 °C for 4h.	EIS, 10 Hz to 1 MHz<br>- SE pellets: φ 10 mm; ~1.2 mm	6.11<br>3.50	0.3<br>0.33
Wet chemical methods				
[4]	Wet chemical synthesis: Li_2S, Li_3PS_4 and LiCl: dissolve in anhydrous EtOH, evaporate at 90 °C under vacuum, heat treated at 200 °C under vacuum	EIS, 100 mHz to 1 MHz, amplitude 100 mHz<br>- SE pellets: φ 12.7 mm; ~0.6 mm thickness<br>- C as blocking electrodes	0.38	0.399
[5]	Wet chemical synthesis: Li_2S and LiCl dissolve in anhydrous EtOH added to β-Li3PS4·3THF/THF suspension; centrifuged to remove unreacted precipitates; evaporate under vacuum, dried at 140 °C for 20 h – **pale yellow product**<br>**- Li_2S, LiCl, Li_3PO_4 impurities**	EIS, 10 mHz to 1 MHz<br>- SE pellets: φ 10 mm; 0.6-0.8mm thickness<br>- SS as blocking electrodes	2.4	-

[16]	(1) Mechanical milling: 600 rpm, 45h<br><br>(2) Ethanol dissolution: EtOH 99,5%, **dark brown solution**, dried at 80 °C under vacuum for 3h	EIS, 100 mHz to 1 MHz<br><br>- SE pellets: φ 10 mm; ~1.5 mm thickness<br><br>- Au as blocking electrodes	1.4 (MM)<br><br>0.014 (EtOH)	0.23<br><br>0.34
[17]	Wet chemical synthesis: Li_2S, P_2S_5 and LiCl: dissolve in anhydrous EtOH (intensive!); stir for 1h; evaporate at 100 °C, heat treated at 450 °C under vacuum<br><br>**- Li_2S, LiCl, Li_3PO_4 impurities**	EIS, 100 mHz to 3 MHz<br><br>- SE pellets: φ 10 mm; 0.63 mm<br><br>- In as blocking electrodes	0.21	0.5
[18]	(1) Mechanical milling: 600 rpm, 40h<br><br>(2) Ethanol and ethyl acetate (4:6) dissolution: evaporated at 150 °C under vacuum<br><br>**- LiCl + unknown impurities**	- EIS, 200 Hz to 100 kHz<br><br>- SE pellets: φ 10 mm; ~0.9 mm; 360 MPa<br><br>- SS as blocking electrodes	0.04 (MM)<br><br>0.06 (EtOH)	-
[19]	Wet chemical synthesis: P_2S_5 dissolved in pyridine; added LiCl; heated to 70 °C and added Li_2S (color change from green to cyan); evaporate at 120 °C under vacuum, dried at 150 °C under vacuum; pelletized and annealed at 550 C for 10 h<br><br>**- Li_2S, LiCl, Li_3PO_4 impurities**	- EIS, 1 Hz to 7 MHz<br><br>- SE pellets: φ 10 mm; ~2.2 mm; 35 MPa<br><br>- In as blocking electrodes	2.6	-

Table S2. Coordinates of atoms used in quantum chemical computations.

no.	atom	X / Å	Y / Å	Z / Å
1	Li	3.187	0.254	6.683
2	Li	6.683	9.616	6.683
3	Li	6.683	6.683	9.616
4	Li	3.187	3.187	9.616
5	Li	0.254	6.683	3.187
6	Li	9.616	3.187	3.187
7	Li	3.187	5.189	1.748
8	Li	6.683	4.681	1.748
9	Li	6.683	8.122	5.189
10	Li	6.683	1.748	4.681
11	Li	0.254	1.748	8.122
12	Li	9.616	8.122	8.122
13	Li	8.122	0.254	1.748
14	Li	1.748	9.616	1.748
15	Li	1.748	6.683	4.681
16	Li	5.189	6.683	8.122
17	Li	8.122	5.189	6.683
18	Li	1.748	4.681	6.683
19	Li	1.748	8.122	0.254
20	Li	5.189	1.748	3.187
**21**	**Li**	Position varies according to Tab. S2.		
22	Li	4.681	8.122	3.187
23	Li	0.464	2.507	5.399
24	Li	5.399	2.507	0.464
1	P	4.935	4.935	4.935
2	P	0.000	4.935	0.000
3	P	4.935	0.000	0.000
4	P	0.000	0.000	4.935
1	S	1.190	8.680	6.125
2	S	8.680	1.190	6.125
3	S	8.680	8.680	3.745
4	S	1.190	1.190	3.745
5	S	6.125	1.190	8.680
6	S	6.125	8.680	1.190
7	S	3.745	8.680	8.680

8	S	3.745	1.190	1.190
9	S	8.680	6.125	1.190
10	S	1.190	6.125	8.680
11	S	8.680	3.745	8.680
12	S	1.190	3.745	1.190
13	S	6.125	6.125	3.745
14	S	6.125	3.745	6.125
15	S	3.745	3.745	3.745
16	S	3.745	6.125	6.125
17	S	4.935	0.000	4.935
18	S	0.000	4.935	4.935
19	S	7.403	2.468	2.468
20	S	2.468	2.468	7.403
1	Cl	0.000	0.000	0.000
2	Cl	4.935	4.935	0.000
3	Cl	2.468	7.403	2.468
4	Cl	7.403	7.403	7.403

Table S3. Diffusion paths of the lithium atom no. 21 listed in Tab. S1 (coordinates in Å).

path	initial point			intermediate point			final point		
	X	Y	Z	X	Y	Z	X	Y	Z
I	4.681	1.748	6.683	–	–	–	3.187	1.748	5.189
II	4.681	1.748	6.683	4.471	2.428	5.399	3.187	1.748	5.189
III	4.681	1.748	6.683	4.471	2.428	5.399	5.399	2.428	4.471
IV	4.681	1.748	6.683	–	–	–	4.681	3.187	8.122

Table S4. Longitudinal relaxation time of ^7Li in seconds measured in temperatures from 25 to 125 °C for samples prepared at temperatures 200, 300, 400, and 500 °C.

T / °C	200	300	400	500
25	0.376(10)	0.33(6)7	0.280(5)	0.289(3)
35	0.324(6)	0.277(4)	0.232(3)	0.244(3)
45	0.273(7)	0.240(4)	0.214(3)	0.218(3)
55	0.229(8)	0.216(4)	0.200(2)	0.202(3)
65	0.204(6)	0.200(4)	0.203(2)	0.202(2)
75	0.191(6)	0.190(4)	0.215(2)	0.216(9)
85	0.181(6)	0.188(4)	0.236(3)	0.225(2)
95	0.186(6)	0.194(5)	0.265(3)	0.259(3)
105	0.198(8)	0.206(6)	0.303(5)	0.296(3)
115	0.216(8)	0.227(8)	0.343(3)	0.347(4)
125	0.237(10)	0.264(6)	–	–

Table S5. The coefficients A, B, and C of the best fit of the function $f(x) = \frac{Ae^{Bx}}{1 + Ce^{\beta Bx}}$ to the experimental data given in Tab. S3; $\beta = 1.6$.

	200 °C	300 °C	400 °C	500 °C
$A \times 10^3$	1,09 ± 0,36	1,63 ± 0,35	1,05 ± 0,15	0,60 ± 0,08
B	3,21 ± 0,11	3,21 ± 0,08	3,14 ± 0,05	3,36 ± 0,05
$C \times 10^7$	4,3 ± 2,3	8,3 ± 3,0	4,7 ± 1,1	1,9 ± 0,4

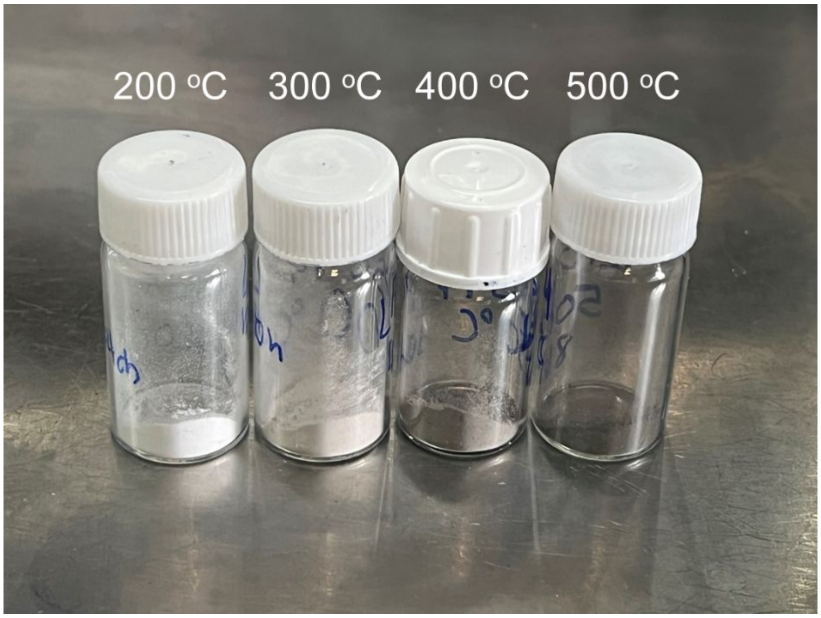

Fig. S1 Picture of LPSC samples annealed at different temperatures.

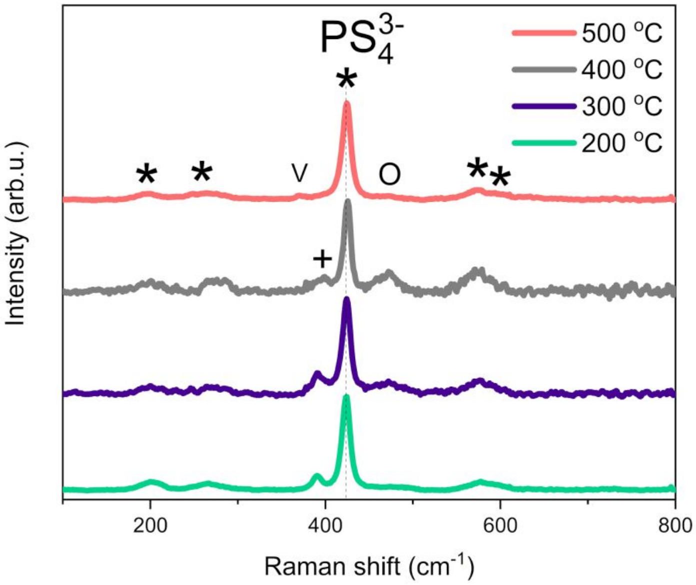

Fig. S2 Raman spectra of all LPSC samples annealed at 200, 300, 400, and 500 °C.

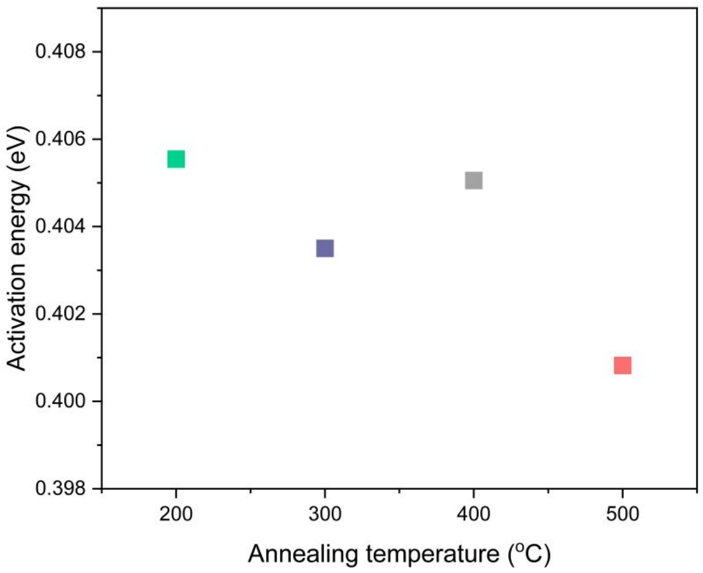

Fig. S3 Activation energies calculated from the temperature-dependent EIS measurements for all LPSC samples annealed at 200, 300, 400, and 500 °C.

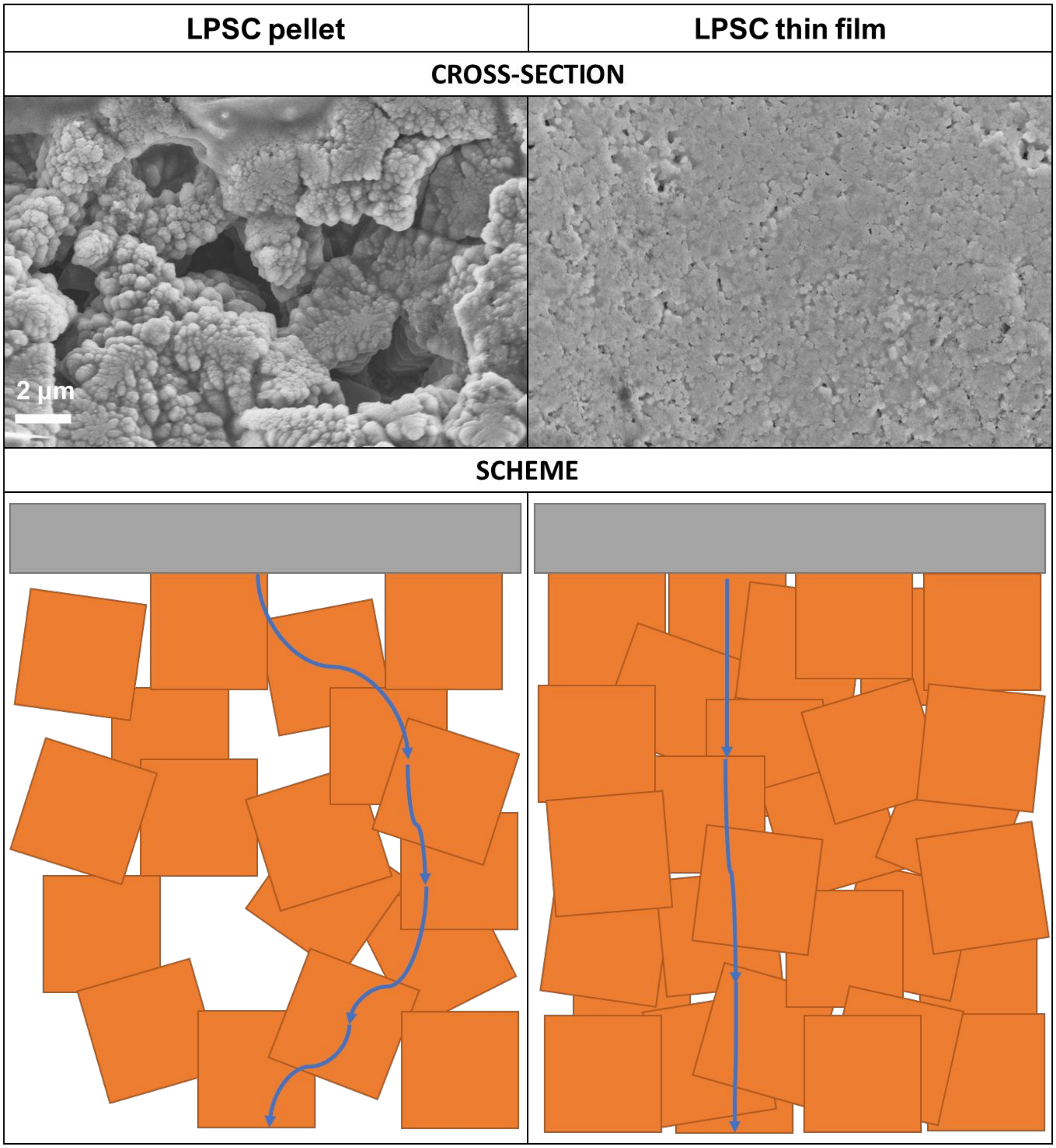

Fig. S4 Schematic of Li^{+} ion conduction pathways.

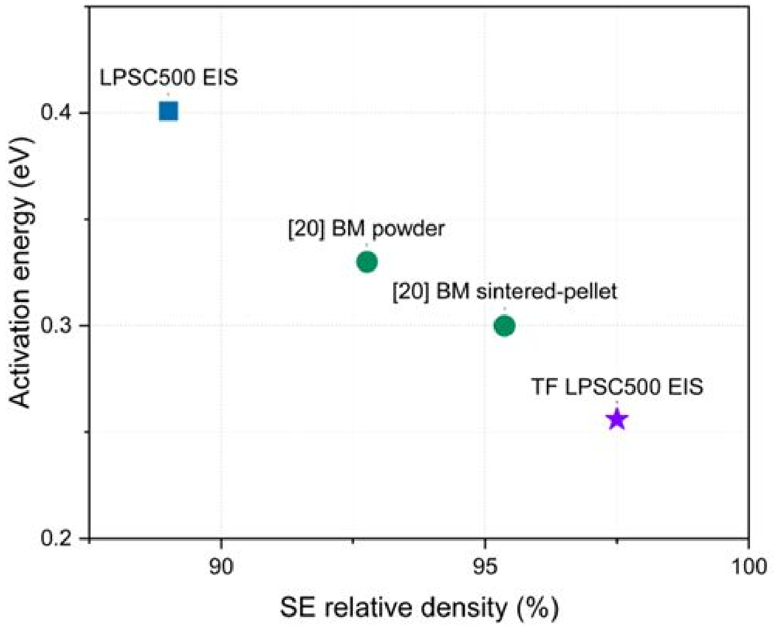

Fig. S5 Experimentally calculated activation energies in relation to relative density of solid electrolyte sample.

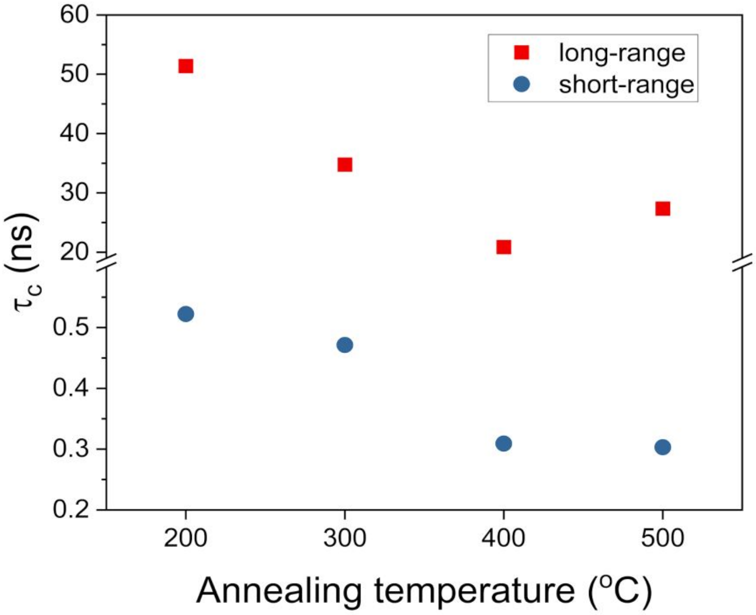

Fig. S6 The autocorrelation time (τ_c) of lithium atoms that fulfills the Arrhenius law calculated for both arms (short- and long-range) of the the temperature-dependence of the longitudinal relaxation rate (T_1^{-1}) function.

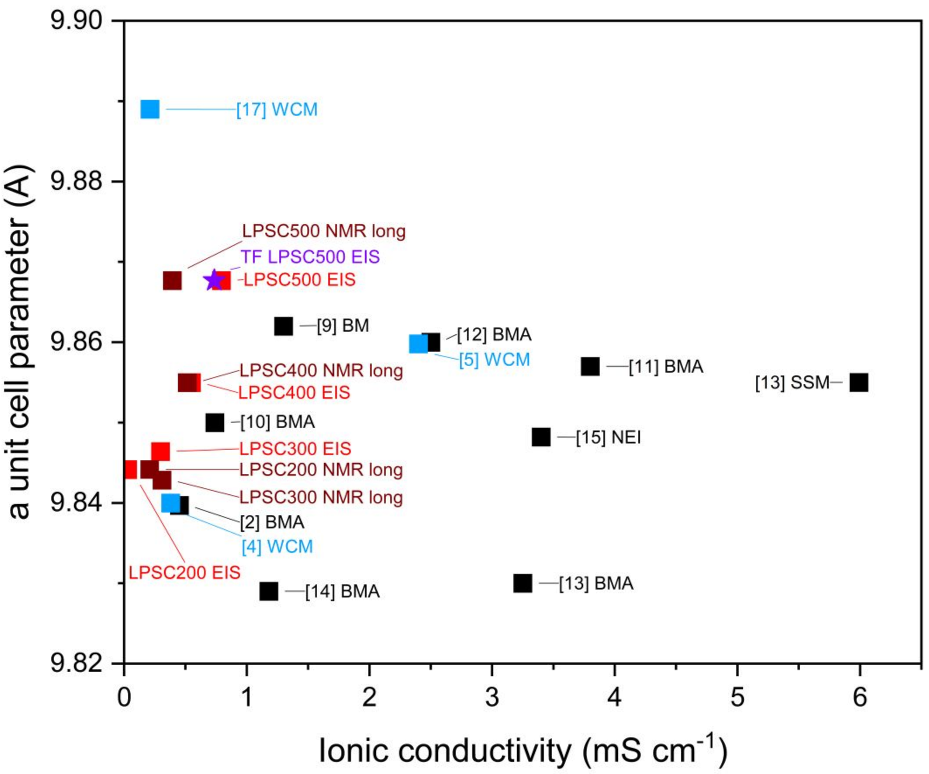

Fig. S7 The comparison of the literature data and our experimental data in terms of *a* unit cell parameter in correlation with Li ionic conductivity. BM- ball milling, BMA – ball milling + annealing, WCM – wet chemical method, SSM – solid-state method.

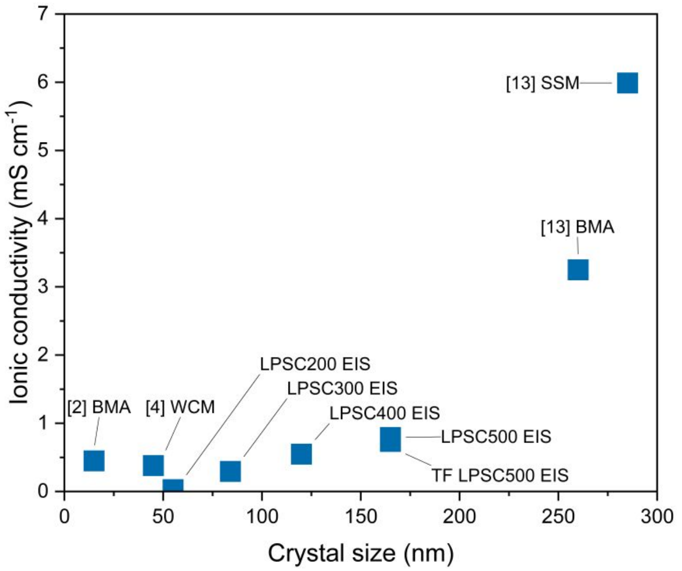

Fig. S8 The comparison of the literature data and our experimental data in terms of crystal size in correlation with Li ionic conductivity. BM- ball milling, BMA – ball milling + annealing, WCM – wet chemical method, SSM – solid-state method.
